# Good clinical but inferior functional outcomes following surgical patellar stabilisation: A prospective study

**DOI:** 10.1002/jeo2.70844

**Published:** 2026-07-09

**Authors:** Oliwer Sygacz, Martyna Jarocka, Tomasz Sacewicz, Jacek Walawski

**Affiliations:** ^1^ Department of Paediatric Orthopaedics and Rehabilitation Medical University Lublin Poland; ^2^ Doctoral School of the Medical University of Lublin Lublin Poland; ^3^ Department of Physiotherapy Fundamentals, Faculty of Physical Education and Health in Biała Podlaska Józef Piłsudski University of Physical Education in Warsaw Warsaw Poland; ^4^ Department of Biomechanics and Anatomy, Faculty of Physical Education and Health in Biała Podlaska Józef Piłsudski University of Physical Education in Warsaw Warsaw Poland; ^5^ Ortosport Lublin Poland

**Keywords:** biomechanical assessment, MPFL reconstruction, MPFL rehabilitation, patellar dislocation, patellar instability

## Abstract

**Purpose:**

To evaluate serial changes in isokinetic knee muscle strength before and after medial patellofemoral ligament reconstruction (MPFL‐R) and to assess whether subjective clinical outcomes reflect objective functional recovery.

**Methods:**

In this prospective study, 39 patients (mean age 25.2 ± 9.0 years) undergoing MPFL‐R for recurrent patellar instability were evaluated preoperatively and at 6 and 12 months postoperatively. Subjective outcomes were assessed using the knee injury and osteoarthritis outcome score (KOOS) and the Kujala score. Objective functional recovery was evaluated with isokinetic testing of knee extensors and flexors at 60°/s and 180°/s using a Biodex dynamometer. Strength and limb symmetry were compared over time between the operated and nonoperated limbs.

**Results:**

KOOS and Kujala scores improved significantly at 12 months compared with preoperative values (both *p* < 0.001), indicating marked subjective clinical improvement. Isokinetic testing showed significant increases in peak torque of both extensors and flexors in the operated limb over time. However, a persistent quadriceps strength deficit of the operated limb compared with the contralateral limb remained at 12 months, particularly for knee extensors. In contrast, hamstring strength deficits decreased significantly, indicating better recovery of flexor strength.

**Conclusions:**

Although MPFL‐R results in significant improvements in patient‐reported outcomes, objective isokinetic testing reveals persistent quadriceps strength deficits up to 12 months postoperatively. These findings suggest that subjective clinical scores may overestimate functional recovery. Objective strength assessment should be incorporated into return‐to‐sport decision‐making following MPFL reconstruction.

**Level of Evidence:**

Level II.

## Graphical abstract

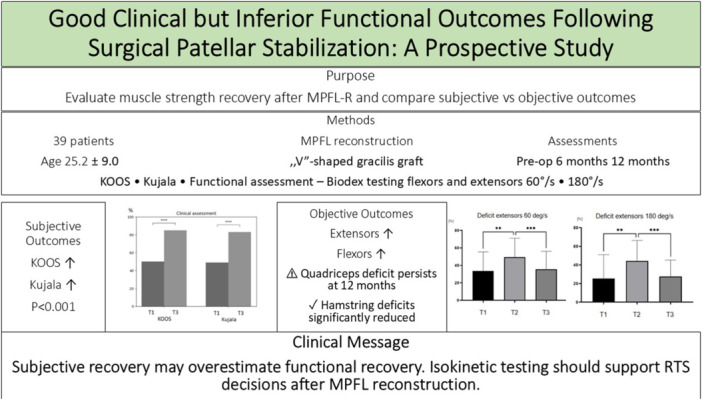

**Abbreviations**
ACL: anterior cruciate ligament
HEP: home exercise program
LSI: limb symmetry indices
MPFL: medial patellofemoral ligament
MPFL‐R: MPFL reconstruction
ROM: range of motion
SD: standard deviation
TT: tibial tubercle
TT‐TG: tibial tuberosity–trochlear groove

## INTRODUCTION

Patellar dislocation is one of the most common knee injuries and the most common dislocation among young adults, making up about 3% of acute knee injuries. The initial traumatic dislocation of the patella typically occurs due to trauma sustained during physical activity or sports. A noncontact knee sprain in flexion and valgus is the primary mechanism behind patellar dislocation, accounting for up to 93% of all cases [3, 13, 18]. Several anatomical risk factors have been identified, including patella alta, trochlear dysplasia, abnormal patellar shape, excessive patellar mobility, variations in medial patellofemoral ligament (MPFL) anatomy, generalised ligamentous laxity, underdevelopment of the vastus medialis obliquus, an increased Q angle, an increased tibial tuberosity–trochlear groove (TT‐TG) distance, excessive femoral anteversion, external rotation of the tibia and valgus alignment [41]. Following the initial dislocation, approximately 96% of patients experience significant damage to the MPFL. No matter which surgical techniques are employed, over 90% of cases result in reported good or excellent clinical outcomes. MPFL reconstruction (MPFL‐R) has shown excellent outcomes and high levels of patient satisfaction, with a low incidence of complications and postoperative failures [24, 34, 43]. In spite of the abovementioned favourable surgical outcomes, the restoration of full knee function and safe return to activity remain as key challenges. Since the implementation of accelerated rehabilitation protocols [25], numerous research [33, 39] suggest that after MPFL‐R procedure as long as 6 months time span is needed for knee joint to recover to the pre‐injury level. However, this view is subject to variance, as some studies have demonstrated persistent alterations in knee joint kinematics and deficits in quadriceps strength lasting up to a year or longer following reconstruction [1, 5, 29].

The understanding of MPFL‐R and the subsequent rehabilitation process is continuously advancing as new evidence emerges. Rehabilitation aims not only at storing stability but also at the recovery of the functional performance, enabling patients to safely return to sports and recreational activities without complications [28].

Therefore, decisions on returning to sports or other intensive physical activity require careful evaluation. In clinical practice, this is typically based on patient‐reported outcome measures such as the KOOS [36, 40] or Kujala [21] scales, as well as objective assessments with the use of knee arthrometers [22]. Both scales are subjective in nature, and measurements of knee laxity do not necessarily reflect the functional performance of the knee joint [9]. Hence, objective biomechanical assessment is essential to provide a more comprehensive evaluation of knee function. This can be achieved using biomechanical tests performed either directly or indirectly. Direct methods involve the measurement of knee joint torques using dynamometers, whereas indirect methods rely on inverse dynamics based on biomechanical models.

The aim of this study was to evaluate serial changes in isokinetic muscle strength around the knee joint following MPFL‐R, with distinct emphasis on identifying and quantifying strength deficits between the operated and nonoperated limbs over time. In addition, authors sought to compare clinical outcomes, as assessed by patient‐reported outcome measures, with objective functional recovery based on isokinetic testing. The hypothesis was that improvements in clinical scores do not inherently correlate with restoration of muscle strength and functional performance. Therefore, this study also aimed to explore the potential discrepancy between subjective and objective measures of recovery, highlighting the importance of functional assessment as a key factor in guiding clinical decision‐making, including return‐to‐sport readiness. There are limited prospective studies that incorporate serial objective functional assessments following MPFL‐R, primarily those evaluating isokinetic muscle strength over time, which caps a comprehensive understanding of the relationship between clinical outcomes and functional recovery.

## METHODS

### Study design

This prospective study included consecutive patients treated surgically for recurrent patellar instability between January 2015 and January 2018. Clinical, biomechanical and patient‐reported outcome measures were assessed 1–2 weeks before surgery and repeated at 6 and 12 months postoperatively. Patients were additionally monitored for a minimum of 24 months to identify late complications, including recurrent patellar instability, range‐of‐motion (ROM) restrictions, pain and patellar fractures.

All participants provided written informed consent and were informed about the study procedures and potential risks. The study was approved by the local Bioethics Committee (Approval No. KE‐0254/148/2014) and conducted in accordance with the Declaration of Helsinki.

### Patient selection

The indication for surgical stabilisation was recurrent patellar instability, defined as at least two documented episodes of patellar dislocation and a positive apprehension test for patellar luxation [2]. Diagnosis was confirmed during clinical examination under anaesthesia. Exclusion criteria included bilateral instability, osteochondral fractures and previous surgery for patellar instability. Preoperative radiological evaluation included measurement of the TT‐TG distance and the Caton‐Deschamps index.

### Surgical intervention and rehabilitation

**Figure 1:**
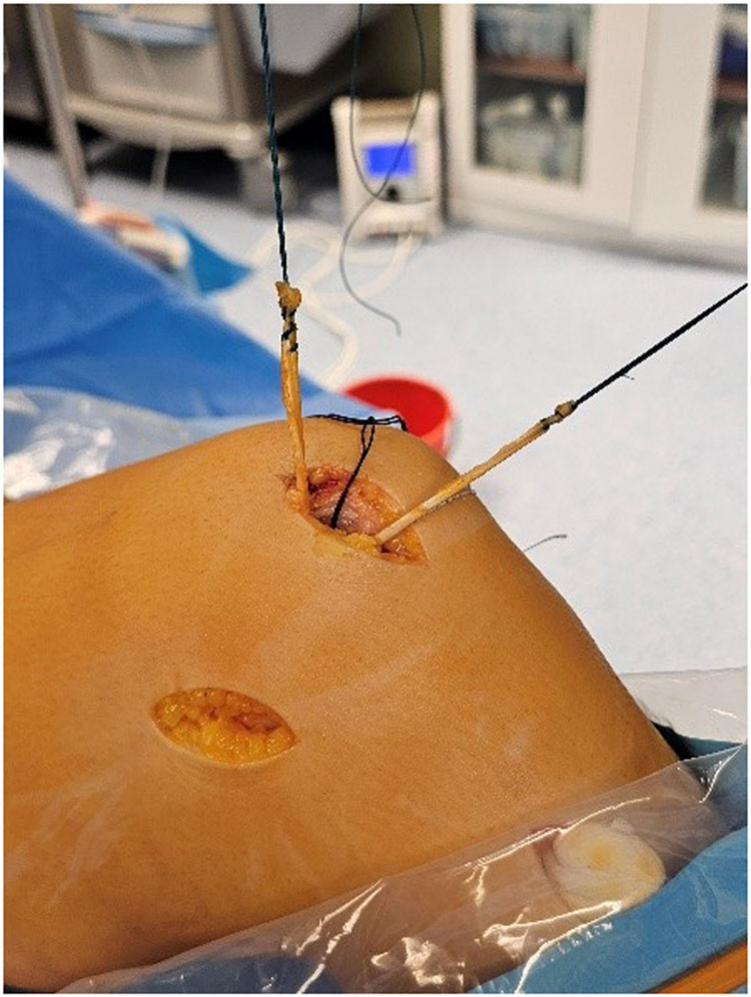
MPFL reconstruction technique with V‐shaped graft arms. MPFL, medial patellofemoral ligament.

**Figure 2:**
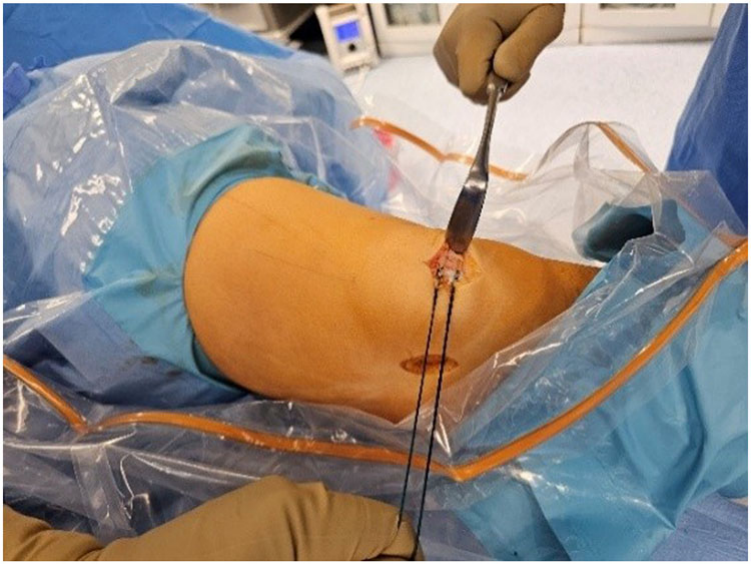
MPFL reconstruction technique with V‐shaped graft arms. MPFL, medial patellofemoral ligament.

MPFL‐R was performed using a gracilis tendon graft configured in a ‘V’ shape (Figures 1, 2). Before reconstruction, arthroscopic examination was performed to exclude concomitant intra‐articular pathology [38]. The femoral tunnel placement was determined using an isometric technique, utilising the adductor tubercle and medial epicondyle as anatomical reference points, with the graft initially secured on the femur. The graft position and tension were assessed throughout the full ROM, aiming for relaxation beyond 45° of knee flexion according to the anisometric principles described by Thaunat [44]. The lateral edge of the lateral femoral condyle served as a reference for proper patellar positioning. In cases of uncertainty, the radiographic technique described by Schöttle was used.

Two 4.5‐mm sockets were drilled into the upper half of the patella, and the V‐shaped graft was fixed using nonabsorbable sutures. If the TT‐TG distance exceeded 20 mm on CT, a tibial tubercle osteotomy was performed to medialize or medialize and distalize the tibial tubercle according to the Caton‐Deschamps index before graft tensioning. Trochlear dysplasia was not surgically addressed.

All patients attended a mandatory preoperative rehabilitation session and received a standardised exercise program and instructional booklet detailing postoperative management, including the PRICE protocol, brace application, crutch‐assisted ambulation and initial strengthening exercises.

Postoperatively, patients wore a long‐leg brace for 4–5 weeks. ROM exercises were encouraged to achieve at least 90° of knee flexion by the end of Week 5. Full weight‐bearing was permitted after 2–3 weeks, although most patients continued using support until Week 5. Patients were advised to attend supervised rehabilitation twice weekly, consisting of manual therapy, individualised exercise progression and guidance regarding the home exercise program (HEP). Since its introduction in 2009, the HEP has been routinely used in the authors' institution for patients with patellar instability and was performed twice daily at home.

Approximately 6–8 weeks after surgery, patients progressed to advanced functional rehabilitation, including cycling, open‐ and closed‐chain exercises and swimming. Return to work or school was generally permitted within 6–8 weeks postoperatively.

### Outcome evaluation

Assessments were performed preoperatively and at 6 and 12 months postoperatively. Although a 24‐month assessment had initially been planned, it was discontinued because of poor patient compliance.

Patient‐reported outcomes included the knee injury and osteoarthritis outcome score (KOOS) [36, 40] and the Kujala Score [21]. Functional assessment involved isokinetic evaluation of quadriceps strength using a Biodex System 4‐PRO dynamometer (Biodex Medical Systems Inc.). Testing was performed by a single experienced biomechanics specialist after a standardised 5‐min warm‐up on a cycle ergometer.

Quadriceps strength of both the operated and nonoperated limbs was assessed at angular velocities of 60°/s and 180°/s according to the study protocol. The 60°/s protocol (5 repetitions) was used as a strength test evaluating maximal isokinetic force, while the 180°/s protocol (10 repetitions) served as an endurance test. Both protocols are commonly used in the assessment of recovery following knee injuries and surgery [4, 16, 26, 27].

The following coding was applied to categorise the timing of the evaluations:

T1: measurement conducted before surgery;
T2: measurement taken 6 months postsurgery;
T3: measurement taken 12 months postsurgery.

### Statistical analysis

Statistical analysis of the results was performed using various descriptive statistics, including arithmetic mean, median, standard deviation (SD), quartile range, minimum and maximum values. Statistical evaluations for the KOOS and Kujala scores were performed using the Wilcoxon test to compare differences in outcomes. The Shapiro–Wilk test was employed to determine whether the distributions of the variables followed a normal distribution. For variables that demonstrated a normal distribution, a one‐way analysis of variance (ANOVA) with repeated measures was conducted, followed by Tukey's post hoc test. Conversely, when a normal distribution was not observed according to the Shapiro–Wilk test, Friedman's analysis was utilised along with Dunn's post hoc test. Statistical significance was assessed using GraphPad Prism version 9.0 software.

During the preparation of this work, the authors used ChatGPT in order to check grammar. After using this tool, the authors reviewed and edited the content as needed and take full responsibility for the content of the publication.

## RESULT

### Patients demographic info

Between January 2015 and January 2018, 68 consecutive patients diagnosed with patellar instability underwent surgical treatment performed by the senior author. Following application of the predefined exclusion criteria (bilateral instability, osteochondral fractures or previous surgery for patellar instability), 47 patients were eligible for inclusion. During follow‐up, 8 patients were lost to follow‐up and did not complete the study protocol. Consequently, 39 patients were included in the final analysis.

The final study cohort consisted of 26 women (66.7%) and 13 men (33.3%). The mean age at the time of surgery was 25.23 ± 9.04 years.

**Figure 3:**
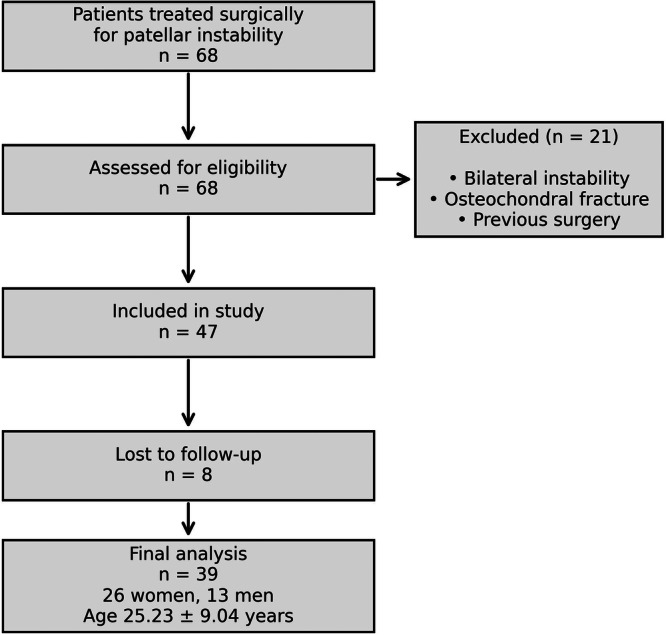
Selection flowchart.

The patient selection process is presented in Figure 3.

### Clinical results

**Figure 4:**
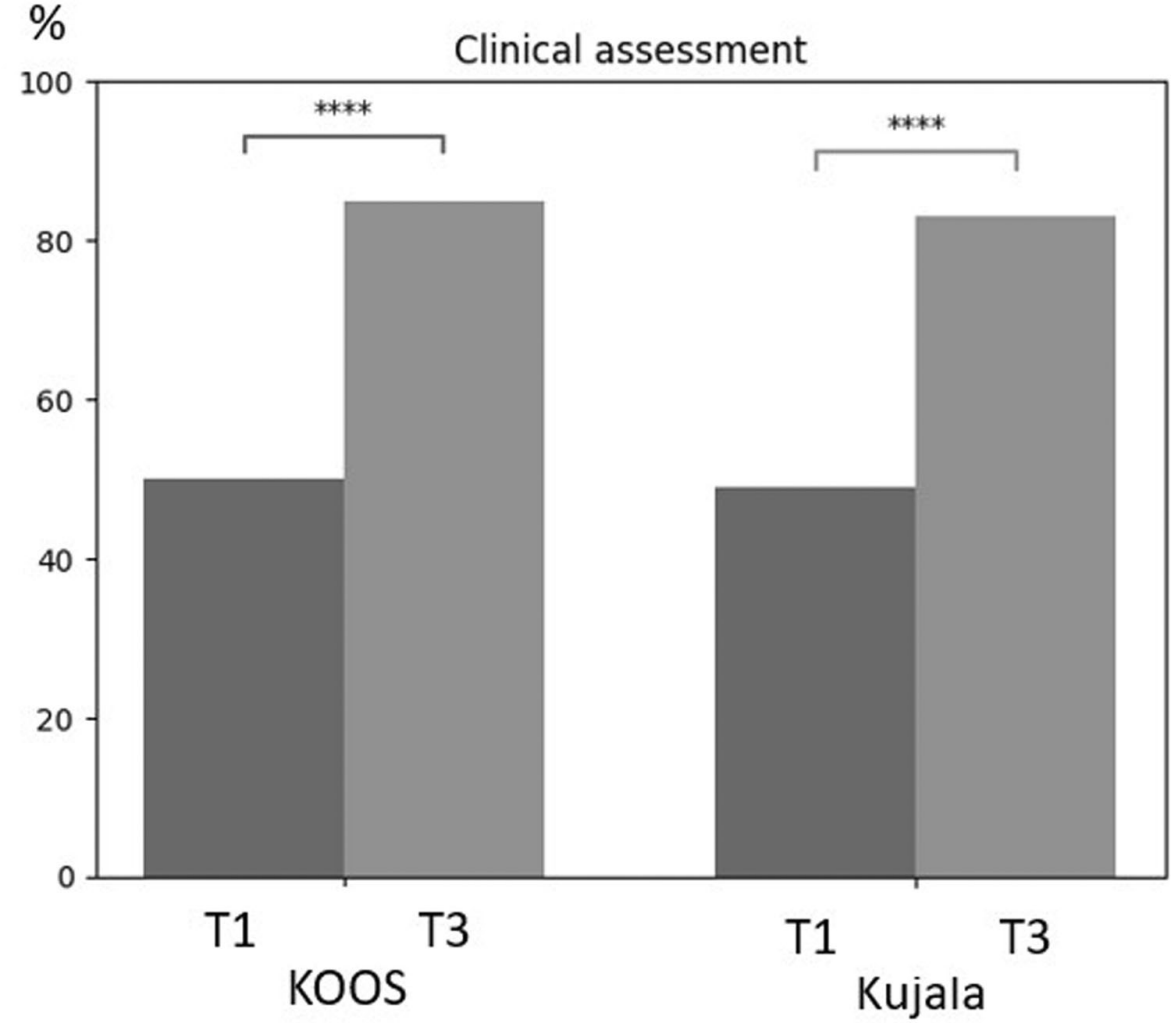
Results of KOOS and Kujala between different stages of the study [Mean] T1‐measurement conducted before surgery; T3‐ measurement taken 12 months postsurgery. ****p < 0.0001. KOOS, knee injury and osteoarthritis outcome score.

Each patient demonstrated notable improvements when evaluated using the KOOS (50.2 ± 15.4 vs. 85.4 ± 8.9, *p *< 0.001) and Kujala (49.2 ± 16.7 vs. 83.1 ± 9.6, *p *< 0.001) scales following the procedure, as compared to their preoperative assessments (Figure 4).

### Complications

One case of patellar redislocation occurred 18 months after surgery, which fell outside the designated timeframe for the functional assessment study. The exact cause of the redislocation remains unclear, and the patient declined further surgical intervention. During the study period, two clinical complications were documented. The first complication involved delayed wound healing, which extended up to 8 weeks postoperatively. The second complication was persistent pain during kneeling, which necessitated the removal of TT screws 14 months after the initial surgery.

### Functional results—Extensors

Results of the mean peak torque of extensors at 60 deg/s are demonstrated below alongside the deficit of the operated limb compared to the nonoperated limb (Table 1).

**Table 1:** Results of the mean peak torque of extensors at 60 deg/s.

Measurement	Operated limb [Nm]	Nonoperated limb [Nm]	Deficit [%]
T1	101.67 ± 47.92	152.38 ± 56.75	33.5 ± 21.9
T2	81.81 ± 47.79	160.00 ± 53.86	49.3 ± 21.82
T3	120.23 ± 59.37	179.48 ± 56.18	35.62 ± 20.57

*Note*: T1‐pre‐operative measurement, T2‐measurement 6 months after surgery, T3‐measurement 12 months after surgery.

**Figure 5:**
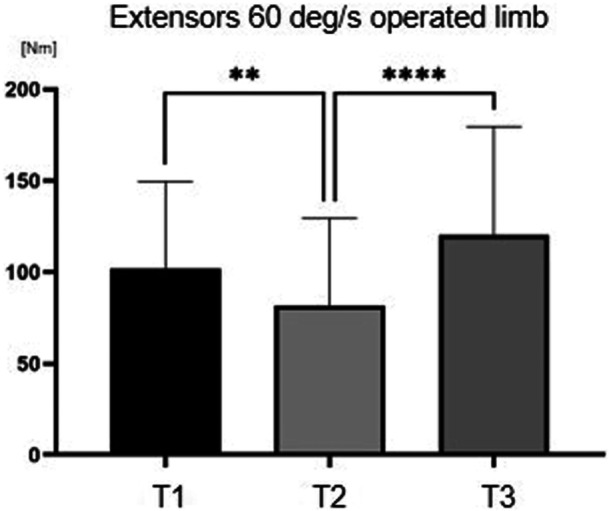
Mean peak torque of extensors at 60 deg/s between different stages of the study of operated limb (2) and nonoperated limb (3). (Mean, SD) T1: measurement conducted before surgery; T2: measurement taken 6 months postsurgery; T3: measurement taken 12 months postsurgery. **p < 0.01, ****p < 0.0001.

Comparing the mean peak torque of extensors of the affected limb at 60 deg/s, measured before MPFL‐R and during the study, a statistically significant difference was observed between T1 and T2 (*p *< 0.01) as well as between T2 and T3 (*p *< 0.0001). There was no statistically significant difference between the preoperative measurement and the measurement taken 12 months postoperatively (Figure 5).

**Figure 6:**
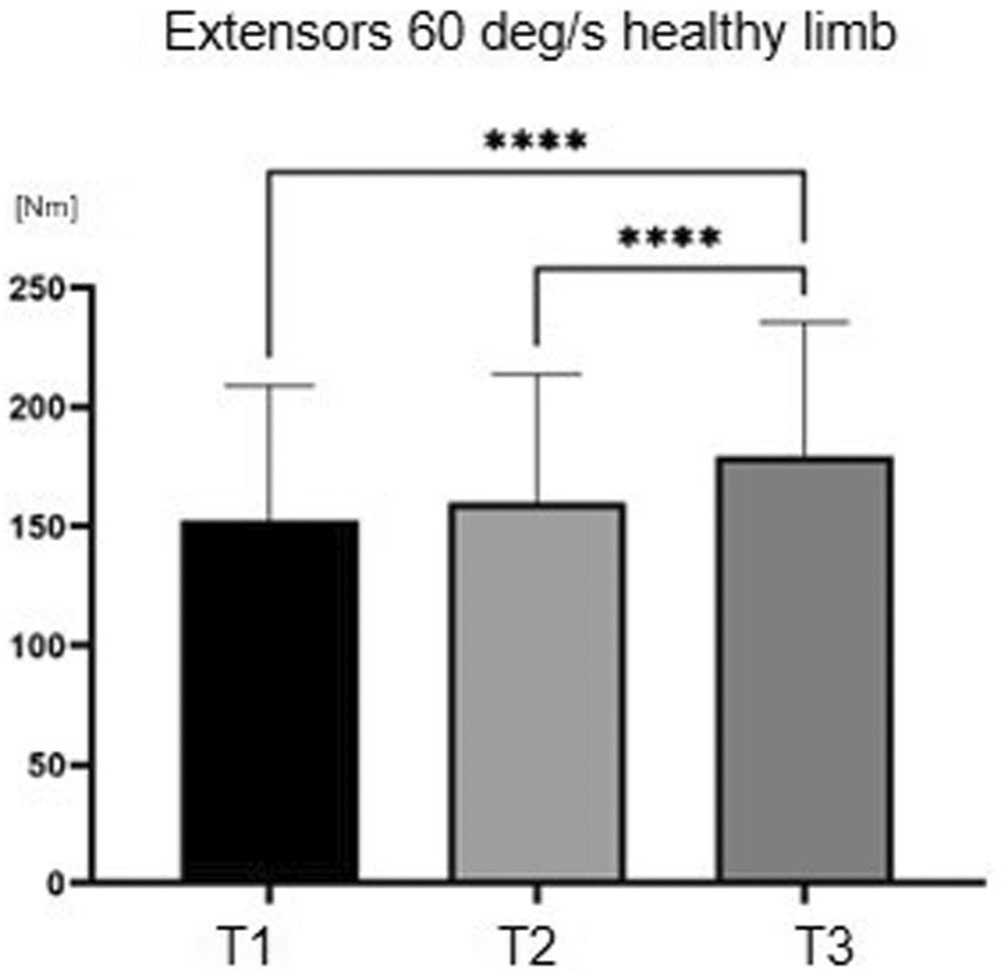
Mean peak torque of extensors at 60 deg/s between different stages of the study of operated limb (2) and nonoperated limb (3). (Mean, SD) T1: measurement conducted before surgery; T2: measurement taken 6 months postsurgery; T3: measurement taken 12 months postsurgery. ****p < 0.0001. SD, standard deviation.

Comparing the mean peak torque of extensors of the nonoperated limb at 60 deg/s, a statistically significant difference was found between T1 and T3 (*p *< 0.0001) and between T2 and T3 (*p *< 0.0001) (Figure 6).

**Figure 7:**
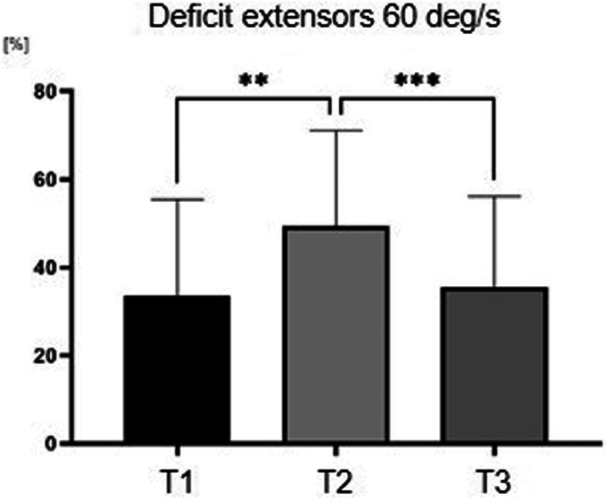
Deficit between operated and nonoperated limb of mean peak torque of extensors at 60 deg/s between different stages of the study (Mean, SD). T1: measurement conducted before surgery; T2: measurement taken 6 months postsurgery; T3: measurement taken 12 months postsurgery. **p < 0.01, ***p < 0.001. SD, standard deviation.

Comparing the deficit between the operated and nonoperated limb in terms of the mean peak torque of extensors at 60 deg/s, a statistically significant difference was observed between T1 and T2 (*p *< 0.01) as well as between T2 and T3 (*p *< 0.001). Notably, there was no statistically significant difference between T1 and T3. The deficit of the operated limb compared to the nonoperated limb persisted (Figure 7).

Below are the findings for the mean peak torque of extensors at 180 deg/s and the deficit between the operated and nonoperated limb (Table 2).

**Table 2:** Results of the mean peak torque of extensors at 180 deg/s.

Measurement	Operated limb [Nm]	Nonoperated limb [Nm]	Deficit [%]
T1	76.83 ± 35.37	100.59 ± 29.31	25.42 ± 25.59
T2	63.83 ± 32.58	112.48 ± 34.56	44.12 ± 22.10
T3	88.57 ± 36.90	120.13 ± 36.35	27.52 ± 17.68

*Note*: T1‐pre‐operative measurement, T2‐measurement 6 months after surgery, T3‐measurement 12 months after surgery.

**Figure 8:**
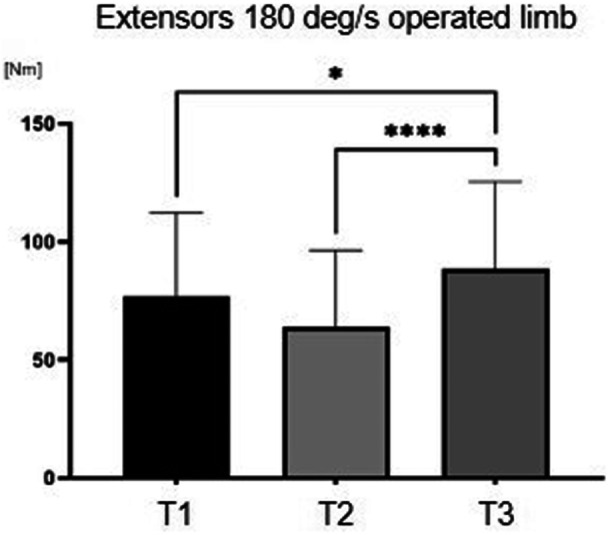
Mean peak torque of extensors at 180 deg/s between different stages of the study of operated limb (5) and nonoperated limb (6). (Mean, SD) T1: measurement conducted before surgery; T2: measurement taken 6 months postsurgery; T3: measurement taken 12 months postsurgery. *p < 0.05, ****p < 0.0001. SD, standard deviation.

When analysing the mean peak torque of extensors in the affected limb at 180 deg/s, measured before MPFL‐R and throughout the study, a statistically significant difference was identified between T1 and T3 (*p *< 0.05) as well as between T2 and T3 (*p *< 0.0001). However, no significant difference was observed between the preoperative assessment and the measurement taken 6 months postoperatively (Figure 8).

**Figure 9:**
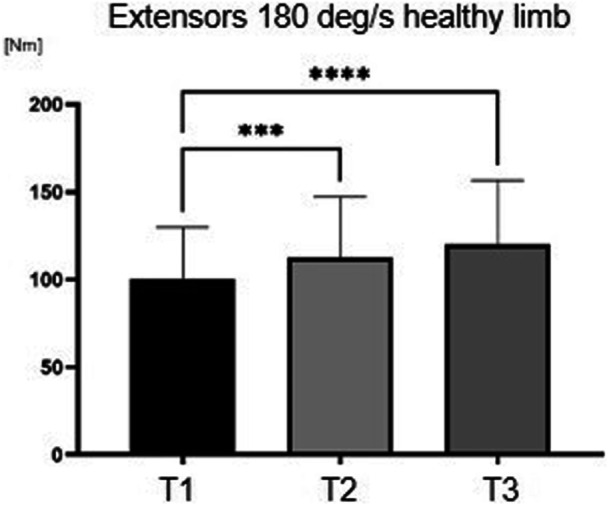
Mean peak torque of extensors at 180 deg/s between different stages of the study of operated limb (5) and nonoperated limb (6). (Mean, SD) T1: measurement conducted before surgery; T2: measurement taken 6 months postsurgery; T3: measurement taken 12 months postsurgery. ***p < 0.001, ****p < 0.0001. SD, standard deviation.

Regarding the mean peak torque of extensors in the nonoperated limb at 180 deg/s, significant differences were noted between T1 and T2 (*p *< 0.001) and between T1 and T3 (*p *< 0.0001) (Figure 9).

**Figure 10:**
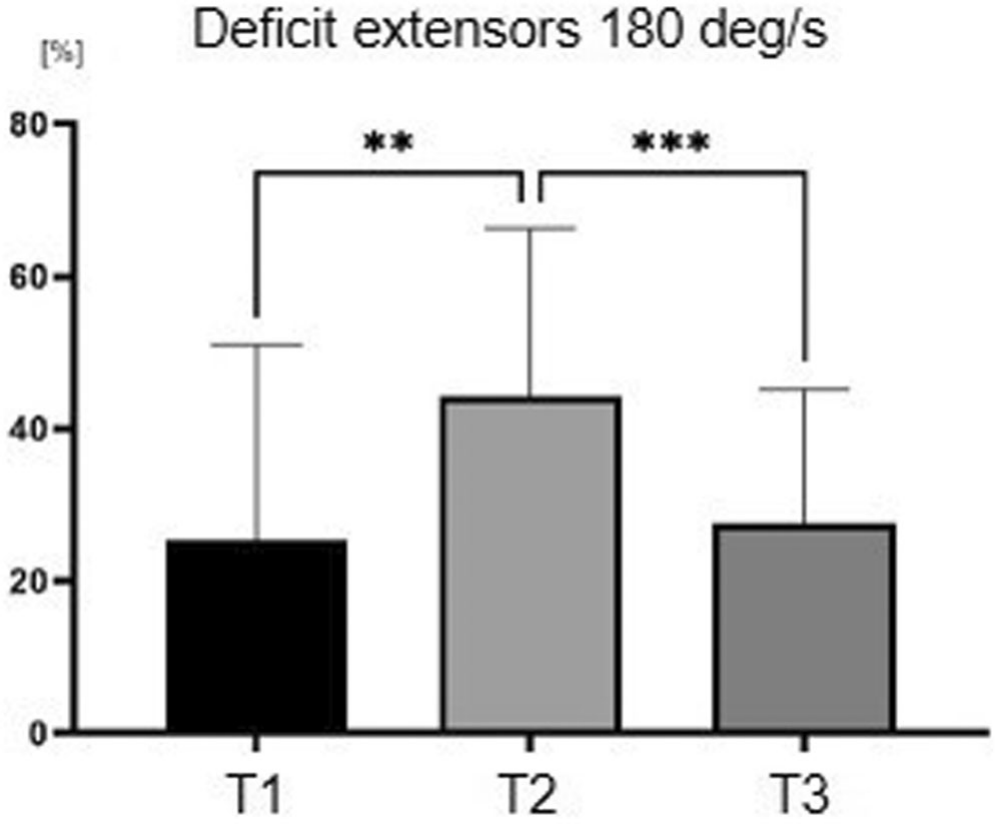
Deficit between operated and nonoperated limb of mean peak torque of extensors at 180 deg/s between different stages of the study (Mean, SD). T1: measurement conducted before surgery; T2: measurement taken 6 months postsurgery; T3: measurement taken 12 months postsurgery. **p < 0.01, ***p < 0.001. SD, standard deviation.

Examining the deficit between the operated and nonoperated limb in terms of mean peak torque of extensors at 180 deg/s, a statistically significant difference was found between T1 and T2 (*p *< 0.01) and between T2 and T3 (*p *< 0.001). Importantly, no significant difference was detected between T1 and T3, indicating that the deficit of the operated limb relative to the nonoperated limb remained present (Figure 10).

### Functional results—Flexors

The study presents findings on the mean peak torque of flexors at 60 deg/s and the strength deficit between the operated and nonoperated limb (Table 3).

**Table 3:** Results of the mean peak torque of flexors at 60 deg/s.

Measurement	Operated limb [Nm]	Nonoperated limb [Nm]	Deficit [%]
T1	56.89 ± 24.66	69.60 ± 22.55	19.44 ± 21.32
T2	64.11 ± 28.18	75.66 ± 25.17	18.89 ± 23.12
T3	82.89 ± 34.18	84.81 ± 28.78	4.74 ± 17.24

*Note*: T1‐pre‐operative measurement, T2‐measurement 6 months after surgery, T3‐measurement 12 months after surgery.

**Figure 11:**
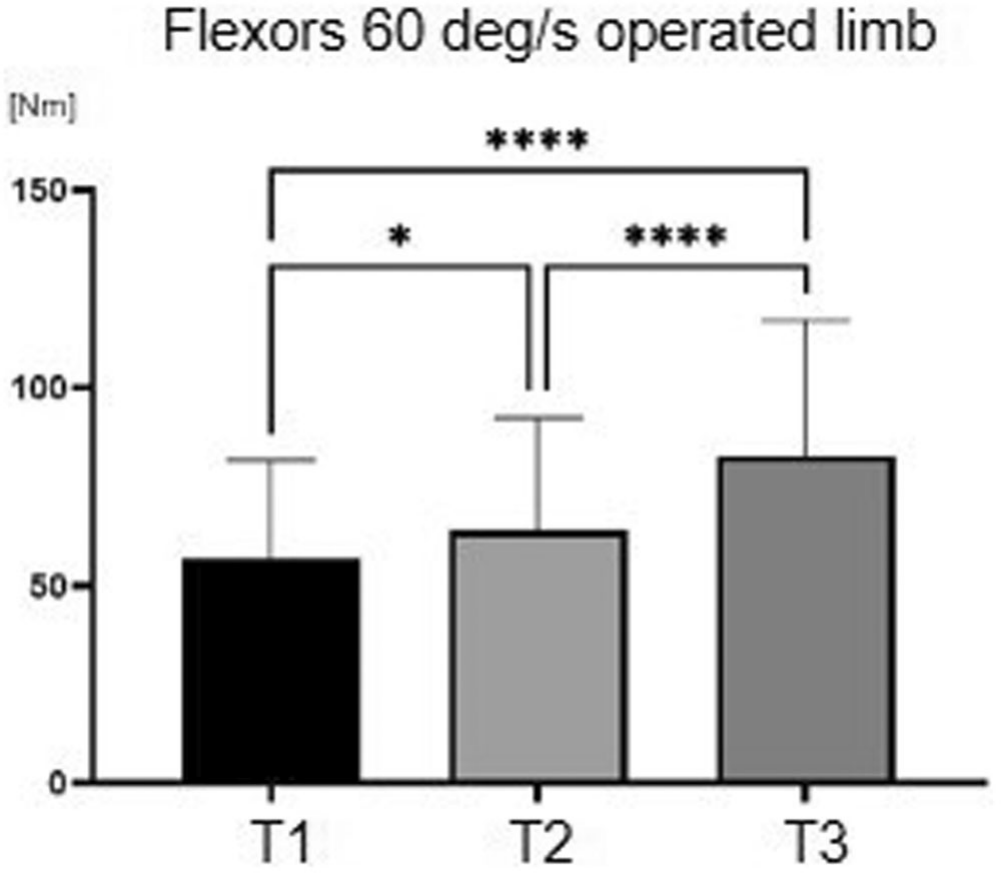
Mean peak torque of flexors at 60 deg/s between different stages of the study of operated limb (8) and nonoperated limb (9). (Mean, SD) T1: measurement conducted before surgery; T2: measurement taken 6 months postsurgery; T3: measurement taken 12 months postsurgery. *p < 0.05, ****p < 0.0001. SD, standard deviation.

Analysis of the mean peak torque of flexors in the affected limb at 60 deg/s, measured before MPFL‐R and over the course of the study, revealed statistically significant differences between T1 and T2 (*p *< 0.05), T2 and T3 (*p *< 0.0001), as well as between T1 and T3 (*p *< 0.0001) (Figure 11).

**Figure 12:**
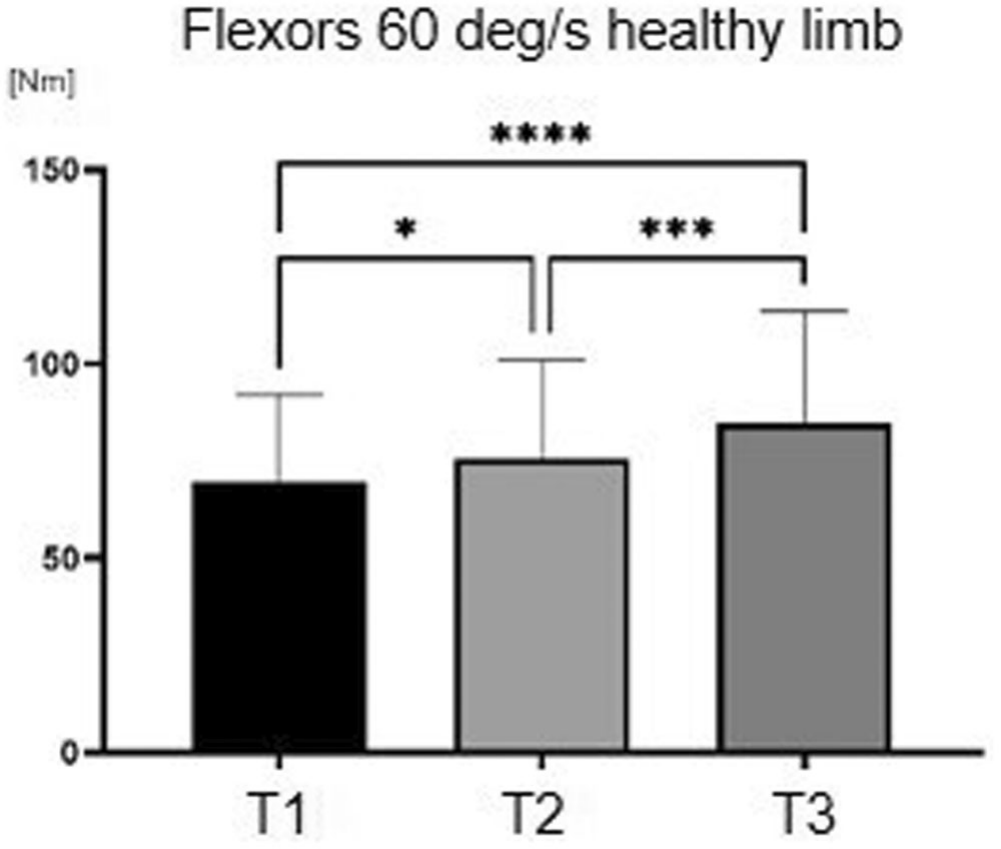
Mean peak torque of flexors at 60 deg/s between different stages of the study of operated limb (8) and nonoperated limb (9). (Mean, SD) T1: measurement conducted before surgery; T2: measurement taken 6 months postsurgery; T3: measurement taken 12 months postsurgery. *p < 0.05, ***p < 0.001, ****p < 0.0001. SD, standard deviation.

Similarly, in the nonoperated limb, significant differences were observed between T1 and T2 (*p *< 0.05), T2 and T3 (*p *< 0.001), and also between the preoperative period (T1) and the 12‐month postoperative assessment (T3) (*p *< 0.0001) (Figure 12).

**Figure 13:**
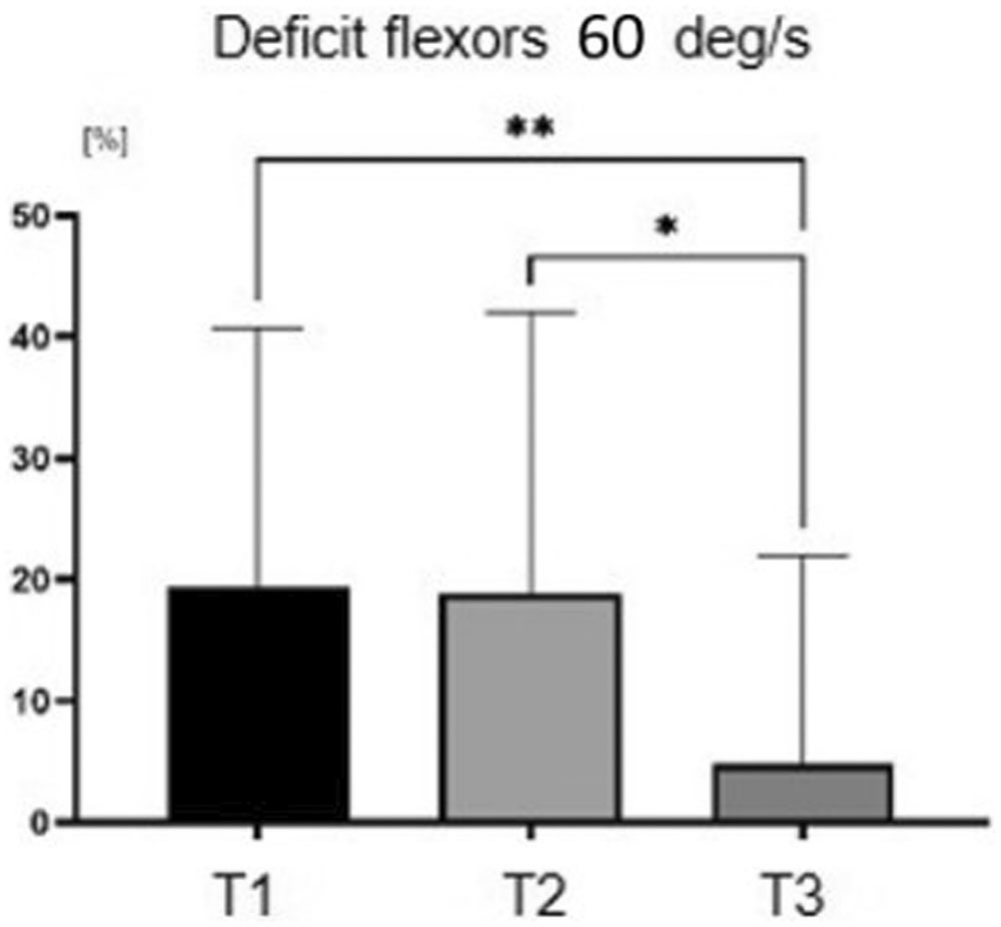
Deficit between operated and nonoperated limb of mean peak torque of flexors at 60 deg/s between different stages of the study (Mean, SD) T1: measurement conducted before surgery; T2: measurement taken 6 months postsurgery; T3: measurement taken 12 months postsurgery. *p < 0.05, **p < 0.01. SD, standard deviation.

When evaluating the deficit between the operated and nonoperated limb in terms of mean peak torque of flexors at 60 deg/s, a statistically significant reduction in the deficit was observed, with differences visible between T1 and T3 (*p *< 0.01) and between T2 and T3 (*p *< 0.05). This indicates an improvement in muscle balance over time (Figure 13).

The study examines the mean peak torque of flexors at 180 deg/s and the strength deficit between the operated and nonoperated limb (Table 4).

**Table 4:** Results of the mean peak torque of flexors at 180 deg/s.

Measurement	Operated limb [Nm]	Nonoperated limb [Nm]	Deficit [%]
T1	45.20 ± 19.97	52.18 ± 16.52	14.99 ± 22.17
T2	53.23 ± 22.62	60.61 ± 21.98	13.91 ± 22.51
T3	62.33 ± 25.21	63.64 ± 22.33	3.93 ± 13.21

*Note*: T1‐pre‐operative measurement, T2‐measurement 6 months after surgery, T3‐measurement 12 months after surgery.

**Figure 14:**
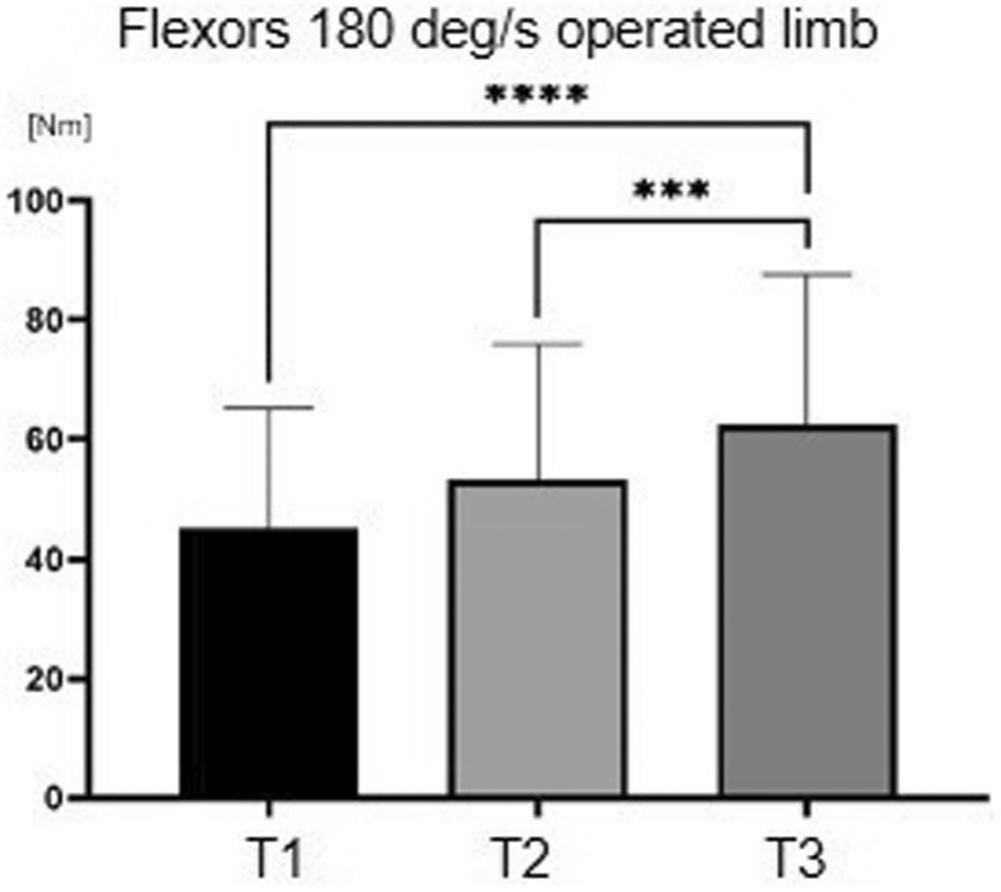
Mean peak torque of flexors at 180 deg/s between different stages of the study of operated limb (11) and nonoperated limb (12). (Mean, SD) T1: measurement conducted before surgery; T2: measurement taken 6 months postsurgery; T3: measurement taken 12 months postsurgery. ***p < 0.001, ****p < 0.0001. SD, standard deviation.

Assessment of the mean peak torque of flexors in the affected limb at 180 deg/s, measured before MPFL‐R and throughout the study, showed statistically significant differences between T1 and T3 (*p *< 0.0001) and between T2 and T3 (*p *< 0.001). However, no significant difference was found between T1 and T2 (Figure 14).

**Figure 15:**
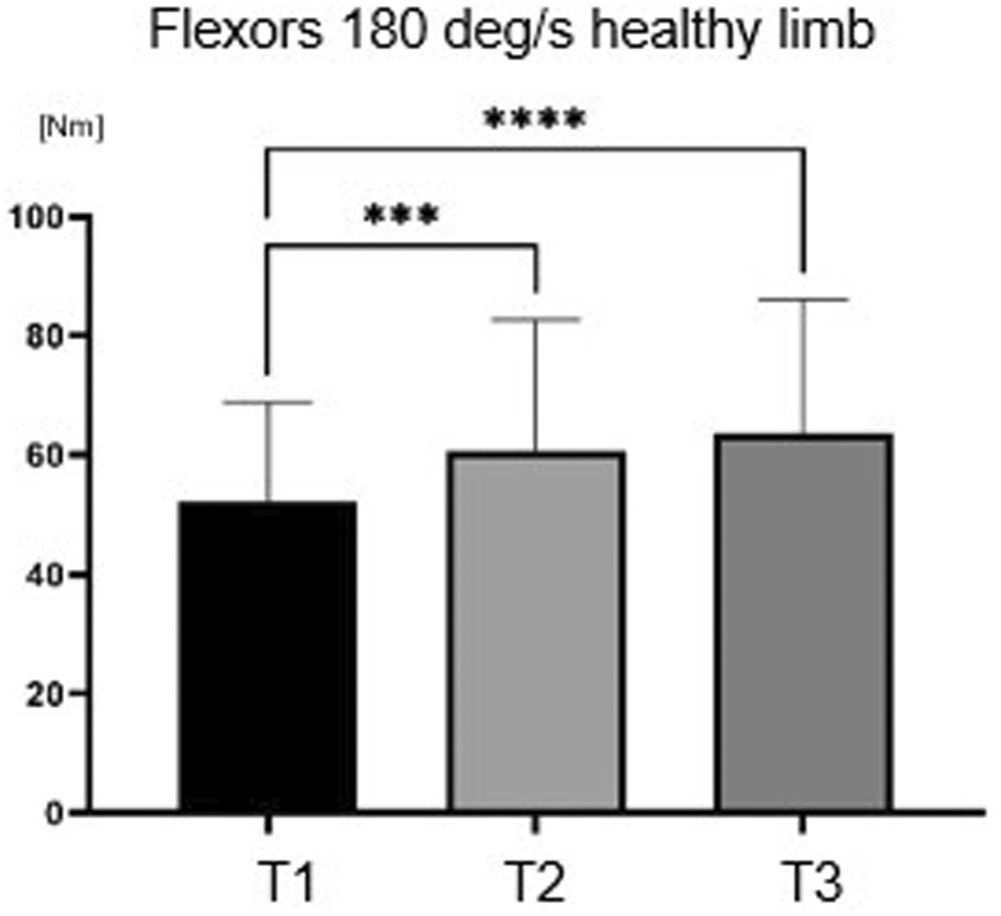
Mean peak torque of flexors at 180 deg/s between different stages of the study of operated limb (11) and nonoperated limb (12). (Mean, SD) T1: measurement conducted before surgery; T2: measurement taken 6 months postsurgery; T3: measurement taken 12 months postsurgery. ***p < 0.001, ****p < 0.0001. SD, standard deviation.

For the nonoperated limb, significant differences were noted between T1 and T2 (*p *< 0.001) and between T1 and T3 (*p *< 0.0001), while no statistically significant difference was observed between T2 and T3 (Figure 15).

**Figure 16:**
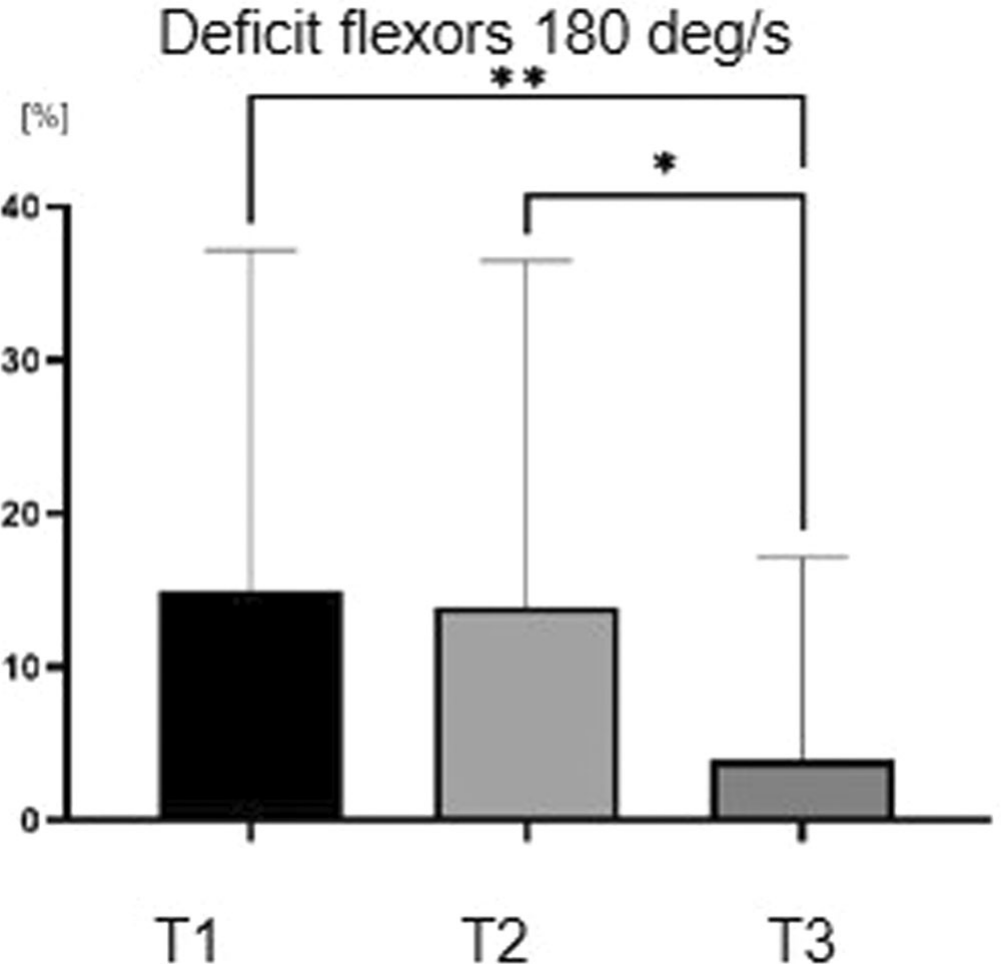
Deficit between operated and nonoperated limb of mean peak torque of flexors at 180 deg/s between different stages of the study (Mean, SD). T1: measurement conducted before surgery; T2: measurement taken 6 months postsurgery; T3: measurement taken 12 months postsurgery. *p < 0.05, **p < 0.01. SD, standard deviation.

Regarding the deficit between the operated and nonoperated limb in terms of mean peak torque of flexors at 180 deg/s, alike findings at 60 deg/s, a significant reduction in the deficit was observed, with differences noted between T1 and T3 (*p *< 0.01) and between T2 and T3 (*p *< 0.05). This suggests a progressive improvement in muscle balance over time (Figure 16).

## DISCUSSION

The study confirms that MPFL‐R leads to significant improvements in patient‐reported outcomes: KOOS and Kujala scores rose substantially at 12 months postoperation (*p* < 0.001). Similar findings were reported by A. Mulliez, who observed a significant improvement in Kujala scores from 53.5 (SD: 22.7) preoperatively to 74.7 (SD: 20.5) postoperatively (*p* < 0.01). Additionally, all KOOS subdomains showed statistically significant improvements (*p* < 0.01). These results were consistent across both patient groups, those undergoing isolated MPFL‐R and those who underwent MPFL‐R combined with tibial tubercle transfer [35]. Matsushita et al. also reported comparable outcomes, demonstrating a significant improvement in the mean Kujala score from 64.6 ± 22.1 preoperatively to 84.7 ± 11.8 postoperatively (*p* < 0.001). Furthermore, the KOOS score showed statistically significant postoperative improvements across all five subscales [30]. These findings are consistent with those of Bremond et al., Carnesecchi et al. and Calanna et al. [6, 7, 8]. These findings reinforce the consistent positive impact of MPFL‐R on patients' subjective perception of knee function and quality of life in daily activities. Similar to the study results, these clinical improvements suggest that the procedure effectively restores a sense of stability and function from the patient's perspective.

Based on current literature, the relatively low complication rate observed in the study may be partially attributed to the older average age of the study cohort and the relatively short follow‐up period. Parikh and Wall reported a complication rate of 16.2% in a large paediatric and adolescent series, noting that many adverse events—including recurrent instability, limited range of motion and patellar fractures—were more common in younger patients and often linked to technical errors or anatomical immaturity [37]. Additionally, a systematic review by Jackson et al. found that overall complication rates following MPFL‐R ranged from 0% to 32.3%, with recurrent instability reported in up to 11% of cases [20]. These findings support the notion that both patient age and duration of follow‐up significantly influence complication reporting and suggest that longer observation in the study cohort may potentially reveal additional late‐onset issues.

The rehabilitation strategies following MPFL‐R are remarkably similar to those after anterior cruciate ligament (ACL) reconstruction, focusing on restoring ROM, neuromuscular control and progressive strength training. Zhang et al. conducted a prospective study comparing open‐ and closed‐chain exercises post‐MPFL‐R and underscored the importance of a structured, phased approach—mirroring ACL rehabilitation protocols—in achieving optimal functional outcomes [15, 23, 31].

Evidence from ACL reconstruction literature provides useful benchmarks for safe return to sport, often based on limb symmetry indices (LSI). LSI thresholds of ≥85%–90% are frequently cited as threshold criteria for return to sport [12]. Despite rehabilitation protocol adherence, patients still exhibit persistent muscle strength deficits in the operated limb longer than 12 months postop [10]. Neuromuscular dysfunctions may persist, suggesting the need for continued monitoring and individualised training to fully restore optimal muscle coordination and joint stability [11]. Moreover, a large cohort study found that approximately 85% of post‐ACL patients failed to reach recommended quadriceps strength/symmetry criteria by 5–7 months postop, reinforcing the need for objective strength [45]. Accordingly, applying similar LSI benchmarks to MPFL‐R patients—aiming for ≥85%–90% symmetry in quadriceps strength—could serve as a more reliable indicator of readiness than clinical scores or elapsed time alone.

Rehabilitation following MPFL‐R places a strong emphasis on the recovery of quadriceps strength, consistent with insights from several studies. A systematic review and meta‐analysis showed significant, persistent deficits in quadriceps strength—specifically, reduced isokinetic knee extension torque—in patients treated surgically or conservatively for patellar instability, lasting up to 3 years postinjury [5]. Furthermore, a focused case series reported that knee extensor LSI improved from 69.2% before surgery to 82.0% after 12 months following quadriceps tendon–based MPFL‐R [19]. This persistent deficit underscores the critical need to prioritise quadriceps strengthening within rehabilitation protocols. Though hamstrings naturally recover faster, data show they still require systematic training. Recent youth MPFL‐R research indicated that many patients did not achieve ≥90% LSI in quadriceps strength at ~7 months, while a higher proportion did meet LSI thresholds for hamstrings [47]. Furthermore, Saper et al. reported that by the time of return to sport, only 44% of young patients achieved a quadriceps strength LSI of ≥90%, highlighting the persistent nature of extensor muscle deficits in this population and the importance of targeted quadriceps rehabilitation prior to clearance for high‐demand activities [42]. These findings suggest while hamstring recovery is important, the true challenge lies in adequately rebuilding quadriceps strength.

Moreover, studies such as Howard et al. highlight that postoperative compliance after MPFL‐R can be suboptimal, particularly among patients with socioeconomic barriers or limited access to rehabilitation services [17]. The study by Metz et al. showed that only a small proportion of paediatric patients achieved high compliance with their prescribed rehabilitation program after knee surgery. Sociodemographic factors such as single‐parent households, longer travel distance and Hispanic/Latino ethnicity were significantly associated with lower adherence to rehabilitation sessions [32].

The above‐mentioned studies are consistent with described findings and support the notion that return‐to‐sport decisions in patients following MPFL‐R should be made with caution, considering persistent quadriceps strength deficits and the need for objective functional assessment.

Although the authors' prospective study had a robust methodology as well as a 12‐month follow‐up, several limitations should be acknowledged. These include a relatively small sample size, absence of a priori power calculation, incomplete functional data at 24 months, variability in rehabilitation adherence and the inclusion of patients undergoing different surgical procedures (isolated MPFL‐R and combined procedures) without separate subgroup analysis. The studies known to us are not conducted in large patient populations. It is challenging to obtain a substantial number of patients who fully complete the study protocol, particularly when participation requires involvement in multiple assessments, follow‐up examinations and biomechanical evaluations [27, 46]. The surgical heterogeneity may have influenced the functional outcomes and represents a limitation of the study. However, in the authors' experience, this cohort reflects a ‘real‐life’ clinical population, particularly regarding the relationship between subjective and objective outcomes and patient compliance after MPFL‐R. Additionally, commonly used outcome measures such as the KOOS and Kujala scales may not fully capture subjective recovery. Although more up‐to‐date assessment tools (e.g., BPII 2.0 [Banff Patellofemoral Instability Instrument 2.0] and NPI Score [Norwich Patellar Instability Score]) have been proposed [14], it should be noted that this study was designed in 2014, before these instruments were widely adopted. Another limitation is that the Kujala score had not been formally validated in the Polish language at the time of the study; therefore, only a Polish translation was used, which may have affected the reliability and cross‐cultural comparability of the patient‐reported outcomes. Research with larger cohorts, standardised rehabilitation protocols, subgroup analyses and longer follow‐up is warranted to further validate the study findings.

## CONCLUSIONS

MPFL‐R resulted in statistically and clinically significant improvements in patient‐reported outcomes, measured by KOOS and Kujala scores. However, isokinetic testing indicated that quadriceps strength in the operated limb remained inferior to one of nonoperated limb also 12 months after a surgery. While subjective clinical recovery appeared satisfactory, functional deficits in muscle strength persisted in some patients' cases

These findings support the hypothesis that subjective clinical scores may not fully reflect functional recovery. Objective strength assessment through isokinetic testing may provide additional valuable insights and could be considered when making a return‐to‐sport decision after the MPFL‐R. This is valid also for individuals practicing a recreational‐level sports. Nonetheless, these results should be interpreted with prudence given the study's limitations, including sample size, lack of power calculation and surgical heterogeneity. Further studies are needed to confirm the above‐mentioned findings.

## AUTHOR CONTRIBUTIONS

**Oliwer Sygacz**: Software; validation; validation; formal analysis; writing—original draft preparation; visualisation. **Martyna Jarocka**: Methodology; validation; resources. **Tomasz Sacewicz**: validation. **Jacek Walawski**: Conceptualisation; methodology; software; validation; formal analysis; investigation; resources; data curation; writing—original draft preparation; writing—review and editing; supervision; project administration. All authors have read and agreed **t**o the published version of the manuscript.

## CONFLICT OF INTEREST STATEMENT

The authors declare no conflicts of interest.

## ETHICS STATEMENT

The study was conducted in accordance with the Declaration of Helsinki and approved by the Institutional Review Board (or Ethics Committee) of Medical University of Lublin (protocol code KE‐0254/148/2014, date of approval: 29 May 2014.

## Data Availability

The original contributions presented in the study are included in the article and Supplementary Materials. The data that support the findings of this study are available on request from the corresponding author. The data are not publicly available due to privacy or ethical restrictions.
